# Editorial: Patient Safety: Delivering Cost-Contained, High Quality, Person-Centered, and Safe Healthcare

**DOI:** 10.3389/fpubh.2020.00288

**Published:** 2020-07-14

**Authors:** Sandra C. Buttigieg, Gianpaolo Tomaselli, Wilfried von Eiff, Vivienne Byers

**Affiliations:** ^1^Department of Health Services Management, Faculty of Health Sciences, University of Malta, Msida, Malta; ^2^Center for Hospital Management, University of Muünster, Muünster, Germany; ^3^Department of General Practice, HRB Centre for Primary Care Research, Royal College of Surgeons in Ireland, Dublin, Ireland

**Keywords:** patient safety, person-centered care (PCC), economic efficiency, ethics, innovation, technology, decision-making

World Health Organization defines patient safety as the absence of preventable harm and the prevention of errors/adverse events in healthcare (1). Despite stakeholders' unanimous consideration that patient safety is a vital principle of healthcare delivery, it remains a concern across health systems worldwide. Across the continuum of healthcare, every process is potentially subjected to adverse events, which may originate from faults/errors in clinical and operational practices, products, procedures, or systems.

This Research Topic reflects the complexity facing patient safety. It also reflects on the challenges involved in delivering cost-contained, high quality, person-centered, ethically sound, and safe healthcare. The contributions project the complexity and multidimensionality of patient safety by highlighting its facets. These include healthcare managers' and leaders' role in prioritizing safety climate for better patient outcomes, and the importance of innovation and new technologies in medicine to drive the patient safety agenda, which in turn leads to the debate of economic efficiency by containing costs through error minimization and waste reduction. The topic discusses the use of complementary and alternative therapies, as well as over-the-counter drugs—which a closer look reveals that these day-to-day practices cannot be ignored. Patient safety also depends on smart decision-making processes and ethical provider-patient relationships. The articles can be grouped into: (i) the role of leadership in ensuring safety climate and clinical performance; (ii) economic efficiency, innovation, and new technologies; (iii) complementary and alternative medicine; (iv) decision-making; and (v) ethics.

Teuma Custo et al. analyze the mediating role of managerial safety practices and priority of safety in the relationship between safety climate and safety performance in intensive care. Their results highlight the suitability of safety procedures, as well as the saliency of the clarity and unambiguity of clinical/managerial information flow. The leader's role is that of being visibly supportive (2) as a safety referent and change agent by prioritizing safety. Safety leaders need to emerge so as to ensure healthcare organizations' ongoing commitment to patient safety.

Three articles deal with economic efficiency, innovation, and new technologies. von Eiff et al. demonstrate that size-specific instrumentation sets contribute to improved operating room's efficiency. Furthermore, Wienert's article debates Health Information Technologies' (HIT) impact on reducing costs while sustaining patient safety. This contribution also considers HIT interventions' adverse effects and implementation failures. In addition, Gong et al. earmark trust, subjective norm, perceived benefit, and persisting habits to achieve providers'/users' confidence in adopting patient-safe online consultation services.

The use of complementary/alternative medicine, and over-the-counter drugs suffers from lack of scientific evidence. Indeed to ensure patient safety, Luketina-Sunjka et al. argue in favor of investigating and regulating their use.

Two articles focus on the process of decision-making so as to manage patients safely. Micieli and Kingston propose an evidence-based flowchart that identifies headache patients needing neuroimaging. These flow-charts, while allowing flexibility at the discretion of professional experts, reduce the degree of variation in case management and enhance decision-making clarity. Lu et al.'s review focuses on shared decision-making, with “Informed consent,” “Surgery,” “Depression,” “Older adult,” and “Patient-centered care” being the most researched areas. The article by Lu et al. also highlights the value of patient-centered care which links very well with the articles by Chan et al. and Tomaselli et al., with the latter emphasizing the shift towardz person-centered care.

From an ethical perspective, Chan et al. show challenges that clinicians face in family practice. Practitioners are becoming more versed at looking for hidden agendas during consultations, which if missed may result in unsafe therapeutic management—in particular involving issues that patients may not be comfortable disclosing spontaneously or at the first encounter. Therefore, by adopting the biopsychosocial model of care, clinicians are better able to reach correct diagnoses, and to identify “hidden” issues similar to that identified by Chan et al., namely family violence, which may indeed be the cause of ill-health. This of course takes the discourse of patient safety to family practice and away from the more commonly researched hospital context.

Tomaselli et al.'s scoping review considers person-centered care from an ethical perspective as distinct from the doctor-patient discourse considered in relational ethics and patient-centered care. The patient is a person and a partner in care with capabilities and resources. It follows earlier work on PCC within corporate social responsibility (3).

In conclusion, this topic portrays patient safety through different lenses and positions itself as an eclectic subject. The importance of this collection lies in the diversity of the contributions which will assist the reader to appreciate the various facets of patient safety. While patient safety was, is, and will remain a topic of immense importance to healthcare, the debate in the future should venture into emerging issues. Future debates should consider patient safety issues in population aging and migration (4), burnt-out professionals (5, 6), technology use (7), point-of-care testing (8), the influence of type of health system on healthcare innovation and therefore on quality improvement (9), and the use of hospital performance dashboards (10) for better visibility of information from bedside to board (11) so as to ensure safety in communication. It is indeed the scope of this research topic to attract the readers' interest and to keep the debate on patient safety alive.

## Author Contributions

SB led the group of editors for this Research Topic. All authors contributed to the article and approved the submitted version.

## Conflict of Interest

The authors declare that the research was conducted in the absence of any commercial or financial relationships that could be construed as a potential conflict of interest.
